# Users’ acceptability of a mobile application for persons with type 2 diabetes: a qualitative study

**DOI:** 10.1186/s12913-019-4486-2

**Published:** 2019-09-06

**Authors:** Astrid Torbjørnsen, Lis Ribu, Marit Rønnevig, Astrid Grøttland, Sølvi Helseth

**Affiliations:** 1 Department of Nursing and Health Promotion, Faculty of Health Sciences, OsloMet – Oslo Metropolitan University, Oslo, Norway; 2 General Practice Research Unit, Department of General Practice, Institute of Health and Society, University of Oslo, Oslo, Norway; 3 Norwegian Centre for E-health Research, Tromsø, Norway

**Keywords:** Type 2 diabetes mellitus, Acceptability, Satisfaction, Patient perception, Self-management, Healthy lifestyle, Mobile apps, Smartphone, mHealth, Qualitative research

## Abstract

**Background:**

The use of mobile health apps is now common in diabetes self-management and acceptability of such tools could help predict further use. There is limited research on the acceptability of such apps: use over time, the factors and features that influence self-management, how to overcome barriers, and how to use an app in relation to health-care personnel.

In this study, we aimed to obtain an in-depth understanding of users’ acceptability of a mobile app for diabetes self-management, and to explore their communication with health-care personnel concerning the app.

**Methods:**

The study had a qualitative descriptive design. Two researchers conducted 24 semi-structured in-depth interviews with adults with type 2 diabetes who had used a digital diabetes diary app for 1 year, during participation in the Norwegian Study in the EU project RENEWING HeALTH. We recruited the participants in a primary health-care setting. The transcripts of the interviews were analyzed using qualitative content analysis on developing themes, which we interpreted according to a theory of acceptability. We used NVivo 11 Pro during the process.

**Results:**

The users’ acceptability of the app diverged. Overall, the responses indicated that the use of a digital diabetes diary requires hard work, but could also ease the effort involved in following a healthy lifestyle and better-controlled levels of blood glucose. Crucial to the acceptability was that a routine use could give an overview of diabetes registration and give new insights into self-management. In addition, support from health-care personnel with diabetes knowledge was described as necessary, either to confirm the decisions made based on use of the app, or to get additional self-management support. There were gradual transitions between practical and social acceptability, where utility of the app seems to be necessary for both practical and social acceptability. Lack of acceptability could cause both digital and clinical distress.

**Conclusions:**

Both practical and social acceptability were important at different levels. If the users found the utility of the app to be acceptable, they could tolerate some lack of usability. We need to be aware of both digital and clinical distress when diabetes apps form a part of relevant health-care.

**Trial registrations:**

Self-management in Type 2 Diabetes Patients Using the Few Touch Application, NCT01315756, https://clinicaltrials.gov/show/NCT01315756 March 15, 2011.

## Background

The foundation of successful diabetes management is education in the disease, promotion of healthy eating and physical activity, as well as the use of medication to regulate blood glucose and prevent complications [1]. Self-management support is necessary to strengthen a person’s ability to live well with diabetes, whether the intervention is behavioral, educational, psychosocial, or clinical [2].

Digital solutions, such as applications (‘apps’) on smart phones, are increasingly in use in health-care in general, and for diabetes care in particular [3, 4]. The number of apps is increasing rapidly and they are easily accessible [5]. Mobile apps are recommended in Norwegian diabetes guidelines to track physical activity in combination with blood glucose registration [6] and in US guidelines as a part of preventing the development of type 2 diabetes [7]. However, the European Society of Cardiology guidelines, in collaboration with the European Association for the Study of Diabetes, recommend multifaceted strategies acting through multidisciplinary teams without mentioning the use of technology [8].

The use of mobile apps can give persons with chronic diseases, such as type 2 diabetes, improved glycemic control, symptom control, and improve their health outcomes in general [9, 10]. Previous research has found that such apps can improve patients’ collaboration with health-care professionals and that good interactions when first diagnosed can increase the benefits of using an app for persons with type 2 diabetes [11–14]. In addition, it is beneficial if the features in the app are tailored to the users’ needs. In this regard, McMillan et al. have suggested that if the glucose and physical activity feedback were visual, it could increase the participants’ motivation for self-management. Use of behavioral change theories to develop the technology could make it more useful [15]. However, there are several barriers for use of the technology in terms of issues related to the app, to the user, and to environmental factors [12, 14, 16, 17].

If an app is to be practical, it needs to be accepted, and the persons targeted must be satisfied. Acceptability depends on a certain level of usability, but it must also facilitate some improvements in self-management [18]. Satisfaction with a tool could lead to changes in behavior even when medical outcomes might be unchanged [19]. Although we know much about how to design apps that are useful, there is limited research on their acceptability [15]. We also know less about the factors that influence their use over time [11]. Further, there is little knowledge about the factors and features in apps that influence self-management for persons with chronic diseases in general, and for persons with type 2 diabetes in particular, and how to overcome barriers to the use of technology in health-care [4, 9, 16, 19–21]. There is a need to find out more about the degree of intensity, and what approaches could enable the technology to be an effective support in health-care [22, 23]. A recent review concluded that diabetes apps might have the potential to reduce glycated hemoglobin (HbA_1c_) levels at a population level, and that these apps can contribute to lifestyle changes [24, 25]. However, exactly how apps contribute to such changes is unclear [26]. Greater insight into how to integrate apps with diabetes self-management care is required [27], informed by the understanding that self-management incorporates cognitive, behavioral, and emotional approaches [28]. This qualitative study aims to contribute to investigations on how diabetes apps can be accepted and used to support daily self-management challenges, and how they could form a part of health-care consultations.

### Aim

To obtain an in-depth understanding of the users’ acceptability of a mobile app for diabetes self-management, and to explore the role of the app in their communication with health-care personnel.

### Framework, acceptability

Acceptability has earlier been described as “whether the system is good enough to satisfy the needs and requirements of the users” [29]. Within the field of technological solutions for health-care, acceptability and satisfaction have been used as synonymous for an explanation of the persons’ perception of a technological device [30]. Patient satisfaction could be defined as “the fulfilment of the expectation or perceived needs of an individual in a particular situation” [31]. Others identify satisfaction as a concept subordinate to acceptability [32] or even as an aspect of usability, which in turn is subordinate to acceptability [29]. Acceptability of health-care devices is necessary for use and «depends on the interactions between a ‘felt need’ for assistance, the recognition of ‘product quality’ – the efficiency, reliability, simplicity and safety of the technology or device, and its availability and cost» [33]. Hirani et al. developed an acceptability questionnaire originally for use in the Whole System Demonstrator study in the United Kingdom and later in the RENEWING HeALTH (European study REgioNs of Europe WorkINg toGether for HEALTH), of which the present study was a part (see below). They took into consideration that the contact with health-care personnel was significant for the users’ acceptability of digital medical devices. They divided the domains into perceived benefit, privacy and discomfort, care personnel concerns, satisfaction, and kits used as substitutions for health-care [32]. Some factors described as part of acceptability could be more important than others, depending on the particular technical solution and the person using the technology. A person’s acceptability of a digital medical device can also be affected by demographic factors such as age, gender and education, expectations, and usability (ease of use). The factors play different roles within individual variations [34].

The concept of *acceptability* is complex, and researchers have described multiple factors. Nielsen distinguishes between social and practical acceptability in his acceptability model, where practical acceptability covers usefulness (utility and usability), cost, compatibility, and reliability [29]. Usability refers to learnability, efficiency, memorability, and error reduction and what is considered subjectively pleasing. Social acceptability is less elaborate as a concept, but reflects a person’s attitudes toward technology. High practical acceptability scores do not necessarily reflect high social acceptability scores [29, page 24]. We have used elements from this model to interpret our findings, allowing an open approach to the concept of acceptability. These are covered in the Discussion section, where the distinction between social and practical acceptability is emphasized.

## Methods

In the presentation of the methods, we have followed recommendations of the Consolidated Criteria for Reporting Qualitative Research (COREQ) [35].

### Design

This qualitative study was part of the Norwegian Study in RENEWING HeALTH: an intervention designed as a randomized controlled trial with this study performed at the end. An evaluation of the process was performed on completion of the trial [36]. We wanted to explore the RCT findings in depth, and to identify and explain variations in the experiences with use of a mobile phone-based diabetes diary app. We therefore applied a descriptive design with semi-structured interviews to acquire more knowledge about the participants’ perceptions of the intervention. We conducted the interviews after the participants had completed the study at the last assessment period, when evaluating intervention effect.

### The randomized controlled trial in RENEWING HeALTH

The Norwegian study in the EU project RENEWING HeALTH was a 1-year, three-armed randomized controlled study (RCT). Inclusion criteria for participants in the RCT were age ≥ 18 years, having type 2 diabetes, an HbA_1c_ level ≥ 7.1%, the ability to use the equipment, and being capable of filling in questionnaires in Norwegian. The parent trial had a three-group comparison (usual care, app, and app/health counseling). This qualitative study included participants from the two intervention groups (app and app/health counseling). Detailed descriptions of the RCT have been published elsewhere [37–39].

The participants included in the two intervention groups were given a smartphone, i.e., the HTC HD Mini® Windows® Mobile 6.5 containing a digital diabetes diary on an app (the Few Touch application [40]), along with a OneTouch® Ultra Easy® LifeScan Inc., Milpitas, CA, USA, which transferred the blood glucose measurements to the app via Bluetooth. The diary also contained functions with the possibility of setting personal goals, and manually registering daily activity and diet. In addition, an encyclopedia with diabetes-relevant information was included in the app. One of the intervention groups received health counseling from a specialist diabetes nurse for the first 4 months as a boost. The purpose of delivering the app and providing health counseling was to enhance the self-management of diabetes. The intervention was provided outside usual care, but the participants were encouraged to show the app to their health-care personnel. Specific instructions as to how often the participants should use the app were not provided since their needs varied. The mobile app had been developed earlier and tested in collaboration with 12 persons with diabetes [40].

### The research group

The research group included four nurse researchers (SH, LR, MR, and AT) and one researcher with a background in technology (AG). More specifically, their competences were: Professor and experienced qualitative researcher (SH): Associated Professor with a PhD and a diabetes researcher (LR); Associated Professor with an MNSc (MR); Assistant Professor with an MSc and being a PhD candidate (AT); and an information and communication technology chief advisor with in-depth knowledge about the technology of the project (AG). Different reflexive accounts were obtained during the qualitative data analysis and interpretations due to the diverse professional backgrounds of the researchers.

### Participants and setting

MR and LR interviewed 26 persons with type 2 diabetes living independently in the north and south of Norway. The study participants were recruited via general practitioners. In addition, some of the participants were followed-up by diabetes specialist nurses or health-care providers in the municipality or at hospital [39]. All participants had a 1-year experience with the use of a diabetes diary app throughout the study. The interviews were performed between May 2012 and March 2013.

#### Participant selection

The participants in the intervention groups were asked to participate in an interview at the end of the randomized controlled trial. They were then asked to participate in the interviews for this study either by telephone, or at the last meeting with the researchers. If they agreed to participate, the interviewers called them to make an appointment and sent them written information in addition to the information and informed consent they had given earlier at first inclusion in the study. Of the 89 participants receiving a smartphone during the study, we assessed the first 50 participants leaving the trial for eligibility. Of these, five refused to participate and 10 were not asked for participation for various reasons (we could not reach them, they spontaneously stated that they would not participate further, or they had bad health). The remaining 35 were willing to attend; however, we were not able to reach seven of them and two were ill. Adequate power to ensure sufficient richness and depth of analysis was reached after conducting 26 interviews [41]. The study had a broad study aim exploring how persons with type 2 diabetes accepted a digital diabetes diary app according to their acceptability with the device. Further, we recruited persons consecutively when they had finished the RCT, without any configuration of the sample, and continued the recruitment until we had a broad scope of participants. The quality of the interview dialogue was good, and the data collected were useful and of interest. Each of the interviews lasted between one, and one and a half hour. Because of technical difficulties, only handwritten notes were available from two of the interviews. We excluded these notes from the analysis, and thus analyzed 24 audiotaped and transcribed interviews in this article.

#### Setting

Of the 24 interviews, MR performed 14 and LR 10. The participants chose where and how they were comfortable to meet. The interviews were performed at the researcher’s office (*n* = 4), at the home of the participants (*n* = 7) or by telephone mainly because of geographic distance (*n* = 13). All the interviews were performed as soon as possible after the participants had completed questionnaires when leaving the RCT study. None of the interviewers had any contact with the participants during the RCT study.

#### Data collection

MR and LR developed a semi-structured interview guide (Table 1) in accordance with the MAST model [42] where patient perspectives regarding the technology provided constitute one of the assessment domains. The interview guide contained open-ended questions and was approved by the project team. This contained questions about living with diabetes and the use of a digital diabetes diary, and the interaction with the app and the others (e.g. general practitioners and family members) concerning their diabetes. In addition, the researchers asked questions about the health counseling offered to those participants in the intervention group who received this. We received rich information about how they perceived the app, but little or no information about the health counseling. However, the participants gave rich descriptions of their struggle to live their life with diabetes as recommended, described in detail elsewhere [43]. The participants gave one interview each. The participants received no remunerations for giving the interviews.

**Table 1:** The key themes from the interview guide

Themes	Descriptions
Primary themes	Satisfaction with, or accept of the use of the mobile app for diabetes self-management Communication with health-care personnel about the app
Specific themes	User experience of the app and its different elements Value of the app to the user Influence of the app in effecting self-management Effectiveness of the app in improving knowledge of diabetes Efficacy of the app enhancing user independence Strength and weaknesses of the app Advantages and disadvantages of using the app The extent to which the app was to liaise with general practitioners and others, and the degree to which data were shared or discussed The effectiveness of the technology in general Use of the study mobile phone as their ordinary phone
Additional themes	The extent to which the participants experienced that their health improved during the study The future of the app and its potential to manage diabetes The identification of participants to target who would most benefit from use of the app Whether or not the individual right to privacy was upheld during the study

### Analysis

#### Data analysis

We used qualitative content analysis to both systemize and analyze the data, and to describe and interpret the meaning of selected aspects of the interviews inspired by the work of Schreier [44]. Qualitative content analysis was used to obtain an overview of the data collected from the interviews. Relevant text segments were selected and similar segments were sorted using coding. Thereafter, their hierarchical order was deliberated to inform the interpretation of themes. As researchers, we moved back and forth between text segments and the transcribed interviews in its origin in order to ensure that our interpretations of the data were in accordance with the context in which they arose during interviews (Table 2). In the analytical process, a combination of a data- and concept -driven strategy was applied [44, page 25]. Initially, a data-driven strategy was selected to support the inductive approach used to develop a coding frame and the themes directly from data (Results section). Later, a concept-driven strategy was applied to interpret the themes according to the acceptability framework (Discussion section). AT read and summarized the transcription of the interviews, selected relevant text segments concerning use of the app, and coded the themes related to the research question. The authors derived the codes from the data material, and identified units of codes within three different themes. The authors held regular meetings while working on the content analysis and discussed the progression and interpretations. We discussed the interviews, the text selection, the coding frame, and themes during these meetings. The data driven themes are presented in the Results section. As a next analytical step, the authors interpreted and discussed the themes within the frame of the Acceptability Model [29] to gain an understanding of the relation between the themes in our findings and the theoretical concept of acceptability. As a final result the authors suggest a broadening of the Acceptability Model. The software NVivo 11 Pro (QRS International) was used to explore relevant text, sort the themes and sub-themes to track some trends in text material arising from each participant’s characteristics, such as age, gender, frequency of use, perceived usability, and duration since diagnosis.

**Table 2:** The stepwise approach used for the data analysis

Steps	Descriptions
Step 1	The entire data were read through to obtain an overview
Step 2	Text segments that were relevant to the research question were selected
Step 3	The text segments were sorted and coded
Step 4	The codes were categorized into themes and sub-themes
Step 5	The interpretations were confirmed by moving back and forth between the data segments and the context in which they arose during the interviews
Step 6	The themes were interpreted by applying a theoretic approach in accordance with the parameters of an acceptability model

#### Reporting

The findings are presented in a descriptive way under three main headings in the Results section, using selected quotations to depict the sub-themes. We removed any repeated and unnecessary words from the quotations to make the sentences complete and understandable, but still retained their meaning. Professional interpreters translated the quotes from Norwegian to English.

### Research ethics

The Regional Committees for Medical and Health Research Ethics in Norway approved the study (REC no 2010/427). The participants had to give their written approval in an informed consent form related to their participation in the main RCT, and they gave their permission to be contacted for query of participation in qualitative interviews when they had finished the trial.

the interviewing researchers gave some additional written information to the participants about the implementation of the interviews, the interview themes, and the interviewers’ contact information. Code numbers were used to replace names in the transcribed interviews and we have removed identifying information. The audiotapes were stored in a locked safe.

## Results

The median age of the participants was 61 years (range 38–79). Eleven of the participants were men and 13 women. The median HbA_1c_ level at baseline for the RCT was 7.6%; 1 year later at about the time of the interviews, it was 7.7%. Median diabetes duration was 12 years (a range of 1–22 years; mean of 11 years). Only nine of the persons used long-acting insulin and three of them used short-acting insulin additionally. Many of the participants reported using the app steadily through the year (*n* = 12), while others used it, but could at times put it away (*n* = 6). Some stopped using it rather quickly after it was obtained (*n* = 6).

**Fig. 1:**
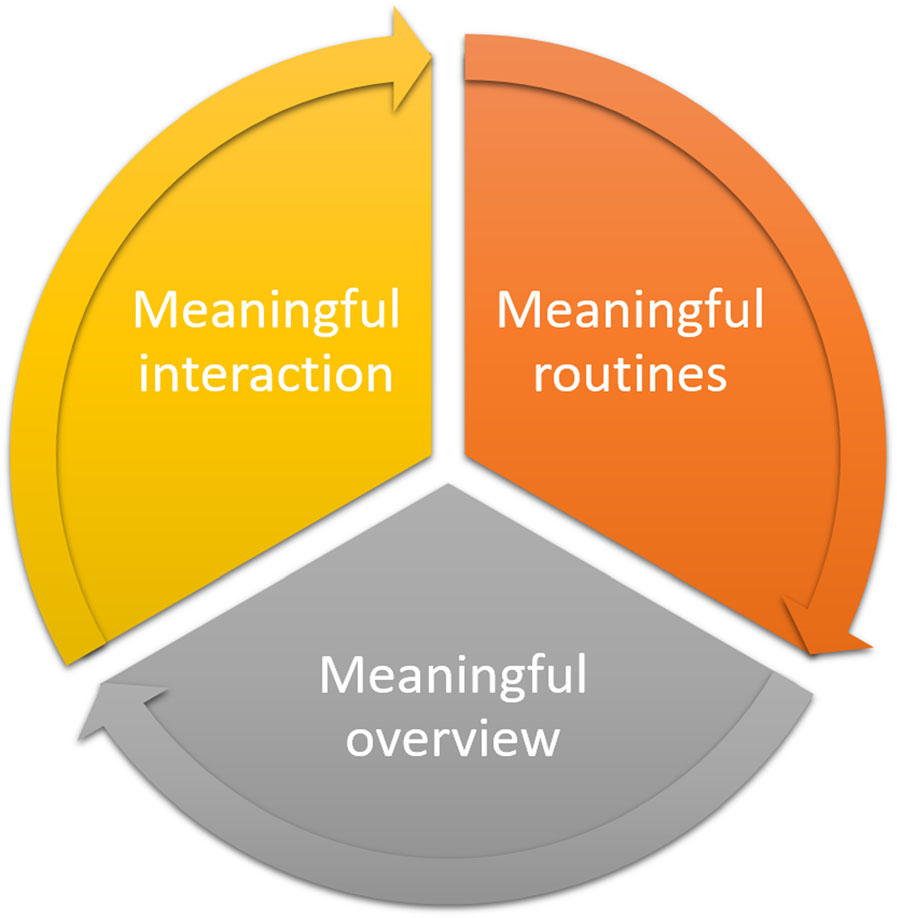
Aiming for a healthy lifestyle supported by the app

In our analysis, we found that an overall theme was that the use of a digital diabetes diary app required hard work, but also that the app could ease the effort in aiming for a change in lifestyle and for better-controlled blood glucose levels. Three themes emerged from the data. Firstly, the app has the potential to contribute to the establishment of *meaningful routines* to measure and store blood glucose levels, diet and activity. Secondly, assuming that this can be achieved, it has the potential to give a *meaningful overview* over their progress and ensure a more balanced blood glucose levels. The third theme was *meaningful interactions* with health-care personnel with or without use of the app. We found that the three themes formed a circular process (Fig. 1).

### Meaningful routines

The participants who used the app had established routines for its use. The participants’ needs and severity of diabetes were diverse; likewise, the routines they established were diverse, but they experienced them to be meaningful for their particular needs. The themes appeared with different sub-themes and with different experiences described by the participants (Table 3). The sub theme *inspiration* refers to the app inspiring the participants to engage in self-management activities. The sub theme *barriers* refers to how the technology itself and in combination with various life issues might lower their inspiration to use the app. The third sub-theme contain suggestions to app improvements supporting routine making.

**Table 3:** Overall theme, themes, and sub-themes

Themes		Descriptions		
Overall theme		Aiming for a healthy lifestyle supported by the app		
Themes		Meaningful routines	Meaningful overview	Meaningful interactions
Sub-themes	Meaningful use	Inspiration: To establish or keep up routines of engagement in self-management activities	Understanding the test results: The person identifies healthy lifestyle patterns by the app data	Decision support: The person made a self-management decision based on app data. Need for health-care personnel’s confirmation
	Barriers and needs for meaningful use	Barriers: To use of the app in real life setting	The need for interpretation: Difficulty to understand the relationship between glucose levels and lifestyle choices	Interpretative support from health-care personnel: The person need help to interpret the app data results
	Suggestions for meaningful app utilization	Routine support: Suggestions on how to use the app	Interpretative support from the app: Suggestions on how to understand the patterns of app data	Alternative support to the app: Reasons against involving health-care personnel and suggestions on other types of health-care support.

#### Inspiration

Using the app could both inspire the participants to establish and give them an obligation to keep up routines in diabetes self-management. Several factors contributed to establishing meaningful routines. Easy accessibility to the app was one of them. The participants reported that it was easy to make notes in the app, the smartphone was always available, and they appreciated the automatic transmission of their blood glucose levels. Another advantage of the application was that it provided a structure. It was easy to organize measures of blood glucose, diet and activity, and easy to read with graphs and charts associated with each part.

*«I’ve never been the type to make notes in a journal and check measurements and diet and things like that. But it was easier to make notes using the phone.» Participant (P)19, female.*

#### Barriers

Even though the app could make it easier to follow planned routines, the participants described some barriers. A major one for conducting a routine was the cost of time and effort. To manually enter measurements such as physical activity and diet in addition to blood glucose was time consuming in a busy everyday life. It could be forgotten easily, or on the other hand, the constant reminder to be vigilant and diligent could be stressful, and choosing not to use the app could represent a relief. For some, poor health may have limited the ability to perform physical activity, and the purpose of using the app routinely disappeared. Another major barrier was the lack of usability of the smartphone, and to some extent the app. The transfer of blood glucose levels did not always work, and the smartphone was small with small buttons and features that sometimes did not work for all as they should. Several of the participants had to use their own mobile phones in addition to the smartphone, which in turn made additional use of time and effort and could hinder their routines for using the app. Some mentioned traveling as a barrier for routines, with changing circumstances, extra costs with use of data traffic and several devices to manage outside their home.

*«If you’re almost never home, you don’t really have much energy left when you do come home, and then you have to gather up the energy to start all over again.» P15, female.*

#### Routine support

The participants had different suggestions about how to be supported in making routines. The app itself could be improved with a more usable technology, freedom of choice and flexibility between devices to make it easier to use, and more features such as reminders and automatic tracking to ease the entering of data. Sending the data to health-care personnel could even be a stronger incentive to establish meaningful routines. To know that the health-care personnel required the data was described as a motivation of importance.

*«I wish there was something that could give me some advice about nutrition and things like that. It didn’t do that at all. It was more of an overview, reminders, and everything related to measuring blood sugar.» P8, male.*

*«It shouldn’t be a problem for the same information to pop up on the doctor’s screen. I need a little push.» P1, male.*

### Meaningful overview

When the participants had found a meaningful routine in using the app, some of them were able to interpret the entered values of blood glucose, and sometimes diet and activity, to find the relationships among them and how they interact, and to understand what to do to balance their blood glucose at an acceptable level.

#### Understanding the test results

The overview that was given in the app made it easier to understand the causality within the different test results in a shorter time, and they were able to find out what they did wrong sooner. Additionally, the overview made it easier for the persons to understand their condition when they had the measures gathered in one place, with easily readable curves, and with a feeling of control.

*«I can see more quickly what I’ve done wrong and how I can do it correctly.» P14, male.*

#### The need for interpretation

Difficulties were encountered trying to identify meaningful data patterns. Not all the participants found the app helpful in interpreting the data and in stabilizing their blood glucose levels. For some of the participants it became too much of a barrier to use. Always needing to be aware of the blood glucose level, and always having to look for explanations in diet and activity were too demanding for some. Even a systematically gathered and meaningful amount of data could be challenging to interpret if the app did not reveal obvious patterns in blood glucose in comparison with other data. The interpretation could possibly be challenging because of different disease courses, with some more complicated than others. In such cases, the use of the app could turn out to be burdensome rather than helpful. Dietary registration was especially difficult to understand, with registration of high and low carbohydrate meals, which could make the data interpretation confusing. Others did not need the app to stabilize their blood glucose; they knew how to stabilize the levels or used other methods such as pen and paper, or possibly, only a blood glucose meter was sufficient.

*«Yes, the [blood sugar] varied. Sometimes it was high and that might be caused by other things – I don’t know. But I did become kind of stressed.» P27, female.*

#### Interpretative support from the app

Interpretation support describes the participants’ suggested added features to support their interpretation of the generated app data. Some of the participants reported additional needs for interpretation support and gave several suggestions for improvements in the app such as additional knowledge about how to control blood glucose levels and how to make healthy choices. Further, the participants requested educational content in the app, such as a manageable menu and more detailed feedback, including on physical activity.

Several of the participants said that the technology did not give sufficient support.

*«You can put a lot more in your phone than if you, for instance, just measure yourself and write down the values. You can put a lot into your phone if you want to, and if you use it properly.» P23, male.*

In addition, the time for when the app was introduced, was a subject of comment among several of the participants. Many of them would have appreciated getting the app at an earlier stage of the disease to enable more benefits from interactions with the app. However, one stated that the app could benefit all persons with type 2 diabetes independent of blood glucose level. Further, it was expected that one should be ready to make changes if one wanted the use of the app to be beneficial. Others stated that the app gave an inspiration to change.

### Meaningful interactions

The participants demonstrated different needs in their communication with health-care personnel thanks to the use of the app.

#### Decision support

Some of them were able to make decisions about self-management of their type 2 diabetes as a consequence of using the app. However, they wanted to discuss the findings with the health-care personnel, make future health-related plans, and obtain verifications of their interpretation. Some of the participants were in an ongoing interaction with health-care personnel, as in this example where they were trying out different solutions and discussing the results.

*«I work hand in hand with my doctor, and we try to find out what’s best. We look at the results together and then we see how things go.» P4, male.*

#### Interpretative support from health-care personnel

Others were unable to interpret the data on the app and expressed a need for more support from health-care personnel, support on understanding how to stabilize blood glucose, and the ability to exchange data within the app with the health-care personnel between consultations, in order to increase the pressure on maintaining a healthy lifestyle. In this context, treatment-related support refers to assistance with the medication taken and the promotion of a healthy lifestyle. One participant suggested the need for more frequent contact by telephone between the consultations: a positive experience from a previous health intervention.

*«Once or twice during the ‘home period’ she calls you and you can bring up whatever problems you have, what you’re feeling, and you try to solve it together.» P14, male.*

Another participant appreciated having the measures on the smartphone because it was not easy to remember them during the consultation, which was easier with the app.

*«Even if you may know that above this much is too high, and below that is too low … what’s good about this is that after a few weeks, you don’t really remember. If you’re wondering about something, you can just take it out and look. You usually have your phone with you.» P24, male.*

#### Alternative support to the app

Some did not show the app to the health-care personnel. Either they did not want to show it or the health-care personnel did not ask for it. They considered that the HbA1c values taken in the consultations gave the necessary amount of knowledge to the health-care personnel. For others, self-managing of diabetes was a minor topic in the consultations. The participants had different opinions and different experiences. Some were pleased with the situation and some asked for health-care personnel with more diabetes-specific knowledge. One recognized that digital solutions could give better health-care in rural areas.

One of the participants attended the project with the aim of reducing his HbA_1c_ level. He used the app daily; it was no effort, and he noted the potential in the app for more functions. However, he expressed a need for a strategy that aroused, or challenged people more. He did not show the app to his general practitioner as he considered it was of no interest:

*«Because it’s the long-term blood sugar the (doctor) looks at» P23, male.*

## Discussion

**Fig. 2:**
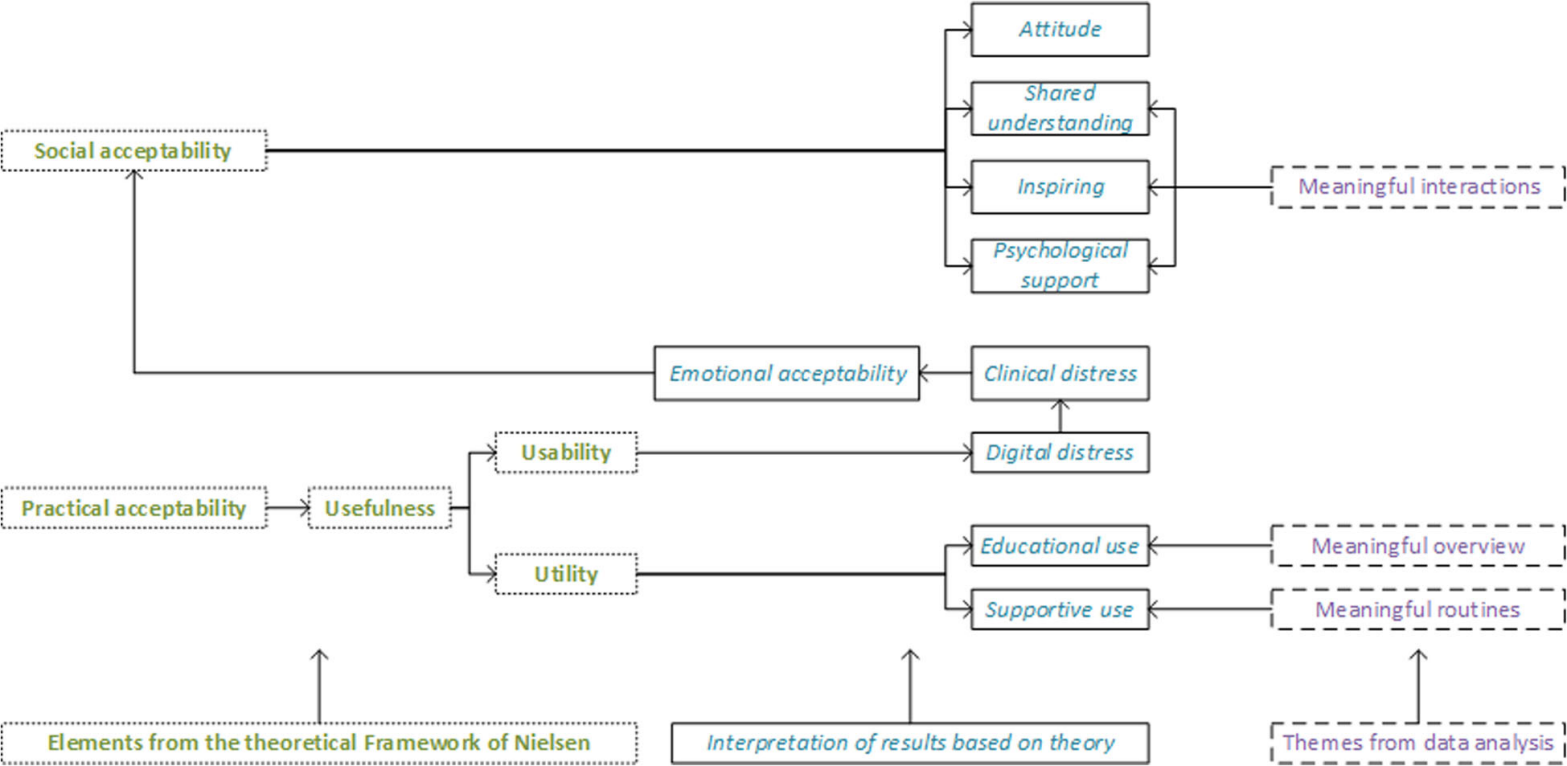
Model illustrating the interpretation of the results (discussion section) and their relationships to the theory (left) and the themes from the data analysis (right)

In this study, we explored users’ acceptability with a diabetes diary app for persons with type 2 diabetes and their voluntary communication with health-care personnel concerning the app. However, the findings were divergent in both the use and the perception of the app. We found that the app in one respect, could lead to stable and meaningful routines and as such an aid for easier living. However, it was also ascertained that having to manually input diet- and activity-related data in the app was demanding, and could represent a barrier for use. Further, the app could also give an overview and be helpful in gaining control over their blood glucose levels in interactions with health-care personnel. Some, but not all participants, used the app in their communications with health-care personnel, but for different reasons. However, there were some barriers for use, such as technological problems, time used on the technology, and motivational and disease issues. Another concern was that some of the participants became stressed by the mobile phone and/or the app and searched for other solutions. Below, we discuss our findings and earlier research, conducted within the parameters of Nielsen’s framework of practical and social acceptability [29], as described previously. Associations between the themes from the results and the acceptability framework is illustrated in Fig. 2.

### The app: practical acceptability

Our findings varied, but showed that for some participants the app could be useful for establishing or maintaining routines and in measuring or implementing a healthy lifestyle. Barriers affected the use of the app to a varying extent. The technology was not always to be trusted. Earlier research has identified multiple barriers for use of technology. Among others, technological challenges could be a major barrier to both the use and the satisfaction of the aids provided [14, 18, 45]. Our findings are consistent with how Nielsen [29] described practical acceptability, where usefulness is one of the components that affects the acceptability of a technology.

#### Usability

Many of the participants described usability as being a challenge: some did not find the app useful for managing routines, while others overcame the challenges, included the app in their routines, and found it useful. The perspective of *usability* was somewhat complex. As an example from an early smartphone, we found that the Bluetooth system was one of the major usability issues when there was a need for support to reconnect the devices. However, the most valued feature of the app was its ability to transfer blood glucose data to it using Bluetooth. Our usability data were less reliable due to recall issues and outdated equipment. Accordingly, compatibility and reliability were considerably reduced after 1 year of app use.

Because of the usability of the app, another point raised was that the time spent using the app was challenging for some participants, while others emphasized that the app was easy to use and available when needed. One of the components of usability described by Nielsen [29] is *efficient to use* which is explained as the time used by an expert user to perform a task with the technology. In our findings, the burden of time using the app did not necessarily have to do with the app itself, but with the burden of the requirements of the app, and the routines of monitoring body and experiences from real life. Other studies have described the burden of treatment where there are multiple demands to care for health. Thus, learning about the disease, continuous self-monitoring, and at the same time struggling with barriers to self-care and diverse health-care provider obstacles, can cause major challenges for individuals [46, 47]. Studies focusing on the prevention of obesity, in which changing diet is a key issue, have highlighted how difficult it is for individuals to change their diet. This can be caused by both internal barriers such as food preferences develop from an early age, and external barriers aroused from how the food industry and associated politics are organized [48]. Our app did not address any of these issues, but could exacerbate the conflicts and difficulties participants meet when the app reminds them about a required change in their lifestyle.

It could be an ethical issue as to whether the app and the smartphone become additional burdens to self-management because of technical challenges. Previous research has pointed out the lack of research on ethical issues related to the use of technology within health-care. Ethical considerations have mostly been associated with the technology itself and its use. Studies have discussed to a lesser extent what the technology is supposed to replace, or what it should be for the users from an ethical perspective [49]. In addition, Korhonen et al. emphasized that from this perspective on digital caring we should know the users’ expectations and experiences, both with the use of the technology and the care [50]. Nevertheless, we found that the use of the app could cause an experience of failure, both in terms of digital data entry and clinical. We could view such *digital distress*, albeit with some limitations, as part of an expected burden when participating in a study aiming to investigate the use of health technology. A certain amount of *clinical distress* is associated with type 2 diabetes [51], but there will be a limit when the clinical distress associated with an uncontrolled disease is caused by using the app, and becomes both unintended and undesirable from a health-care perspective. We should have addressed the crossing of this limit all the way through the year of the study. With newer technology, it will be possible to include awareness, of both digital and clinical distress, but it could require other kinds of self-monitored registrations. However, this would impose additional burden on the users and require a systematic digital follow-up by health-care personnel.

This emotional aspect of acceptability (Fig. 2) has not been adequately described in Nielsen’s model. Thus, we considered the matter to be one of usability. However, the assumption made was that social acceptability is threatened by emotional distress, both with respect to attitudes and psychological support.

#### Utility

*Utility* is a part of practical acceptability, as mentioned previously [29]. Based on our findings, we suggest two different aspects of use: *supportive use* and *educational use*. Supportive use is when the app contributes to establish and maintain diabetes self-management routines, and educational use is when routine use leads to new understandings of the relationships between diet, activity, and blood glucose levels. National standards for diabetes treatment in the US, lists both diabetes self-management education and support as obligations for health-care personnel in their care for persons with type 2 diabetes. Education is the process to facilitate the knowledge, skills, and ability for diabetes self-management while support involves a more informal ongoing assistance to implement and sustain the needed behavior. Such support could be both behavioral, educational, psychosocial, and clinical [2].

Supportive use of the app in our study did not necessarily lead to new knowledge, but was helpful in maintaining a desired lifestyle. The users valued different features to solve different tasks such as storing blood glucose measures or motivating physical activity. We found educational use of the app in our data to some extent by a systematic gathering of lifestyle measurements that the app presented in an interpretable way. Within the app, we had written material as practical examples of use (tutorials), short and longer texts of relevant facts, in addition to the bestowment of “smileys” as rewards. Based on the interviews, it was assumed that neither of these features assisted learning, as only a few participants mentioned that they valued any of them. What was experienced as useful was the linking of blood glucose measurements to other influential factors. Other studies have emphasized that there is a need for apps providing diabetes education, and not apps with only single features [4, 52, 53]. Earlier research on “e-learning” and pedagogy has suggested the effectivity of experimental learning coupled with competent tutoring. The learners are in control of the learning process with help from a tutor [54]. Sharples et al. demonstrated that this could involve parallel learning processes, both digital and interactional, such that the digital process can support interactive learning, and that they become woven together. Control, context and communication could be necessary conditions for learning [55]. From this perspective, we could explain the educational use in our study as experimental. The written material and practical examples were of less use, but communication with the health-care personnel was a necessity for optimal learning, where the digital use of the app was a facilitator.

As a conclusion from our discussion of practical acceptability, we found challenges for all participants, but some found the app useful. Nielsen divided the term *usefulness* in terms of practical acceptability into two components: *usability* and *utility*. Usefulness is the app’s capacity to make the users reach a goal; utility is how the features in the app give an opportunity to perform the desired tasks, and usability is how easy it is to use the features [29]. In our study, it seems that if the users experienced utility and the app could meet a need for supportive or educational use, it was accepted, even when usability came to be a challenge. As we found in our study that the app led to different uses and covered different needs and creativity in the use, and as the participants expressed a wish to use one, it seems that an app that considers the need for different kinds of use - both supportive and educational - could eventually stand with some lack of usability.

### The app: social acceptability

All our participants volunteered for the study and knew about the diabetes diary app intervention. Therefore, we could assume that to some extent they might have intended to use the app from the start. They had a basic attitude that the app was going to be of benefit, and as Nielsen described *social acceptability* as an attitude toward technology [29], we anticipated that the app would be socially accepted initially. However, after a year of use, attitudes diverged among the participants. While practical acceptability failed for some of the users, others found that in various ways the app could enable them to establish routines and thereby improve management of their diabetes. When the users reported the app to be inspiring, we interpreted this as an attitude toward the use of the app, and an expression of its social acceptability. We need to question whether the participants accepted the app as itself, with its technological possibilities, or with certain reservations, such as the importance of additional support.

Some users in our study suggested the app as a tool for communication with health-care personnel, either directly or implicit. Some users envisioned the app to cover a need for self-management support: either as an external motivation for establishing routines where the health-care personnel were actively involved in use of the app, or a more withdrawn role where experience from the app formed the basis for greater understanding through conversations during consultations. As such, the social acceptability of diabetes apps in health-care would depend on a shared understanding of the app between the persons using the app and the health-care personnel. Only a few of our participants used insulin, even though the median duration of their diabetes was long (a median and mean of 12 and 11 years, respectively). Measurements and recording of blood glucose levels was the highest valued feature in the app and a necessary part of its educational use, but in addition, we found that the participants suggested that the use of self-monitoring would be especially important when recently diagnosed with diabetes. However, with self-monitoring of blood glucose levels, earlier research on diabetes management showed lack of evidence on the benefit of self-monitoring glycemic control for persons with type 2 diabetes not using insulin after their first year with their disease [56]. In addition, there is a risk of treatment overuse by both general practitioners and their patients [57]. The guidelines recommend that self-monitoring should be considered in certain disease phases. Patients can benefit by determining how diet and activity affect blood glucose levels at diagnosis, when they are not achieving their treatment goals, and when medication changes or treatment with an intensive insulin regiment are needed. Health-care providers should make decisions about medication in collaboration with the patient, according to personal needs and self-management goals [6, 58, 59]. Health-care personnel might hesitate to recommend broad self-monitoring because there is a lack of evidence on effect. However, social acceptability of the use of apps in self-monitoring could depend on shared understanding between the patient and their health-care personnel of the benefits of such monitoring.

Hirani et al. extended the concept of acceptability and asked for the users’ attitudes toward the app to be a substitution to face-to-face consultations with health-care personnel [32]. Our findings suggest the opposite: that the use of the app in diabetes self-management could represent a valuable contribution, but not a substitution for consultation. When US standards for diabetes treatment initially described the strategy of giving diabetes self-management education, support was not a part of the claim for better treatment. The earlier focus on education lacked the psychosocial aspects of treatment, and diabetes self-management support was introduced as an addition to secure this perspective [2]. Other research has indicated that social interactions are essential to learning processes [60]. The lack of psychosocial support within the app in our study might explain why the users emphasized the need for support from health-care personnel with knowledge of diabetes care. This is in line with other findings suggesting that if such mobile health (mHealth) interventions are to be successful they will require active participation from patients and health-care personnel [24, 25, 61].

In conclusion, the app could have a positive influence on both practical and social acceptability, in terms of the ability to be time efficient, interpretable, adaptable/adjustable, inspiring, and communicative. However, the ability to understand the influence of multiple self-management efforts (which constitutes a type 2 diabetes lifestyle) could be an essential addition to the app. Collaboration between the person with diabetes and competent health-care personnel would also remain an anchor in the basis for treatment, where the app still could be acceptable as a valuable tool used for both supportive and educational purposes. Our adaption of Nielsen’s acceptability is depicted in Fig. 2.

### Future perspectives

In future mHealth research, with the current technology we are on our way to secure practical acceptability in self-management technology. The main issue will be the utility of the system and the social acceptability from the perspective of the users and the health-care personnel, and the personalization of the interventions to the user. Milani and Franklin emphasize that when using artificial intelligence, it is possible to design algorithms aimed at chronic disease management and expert systems. This can enable an overview over a large amount of information, including the user’s own demographic and clinical data as well as personal preferences. Based on this, it is possible to receive even more tailored feedback than with previous interventions [62]. Our findings emphasize the importance of interpersonal and qualified support, and the risk that the technology might cause both digital and clinical distress. With complex systems, further research could develop feedback logarithms and possibly replace nuances in momentous face-to-face meetings with health-care personnel.

### Strengths and limitations

We have based our findings on the use of a digital diabetes diary. Our findings might be of interest not only for persons with type 2 diabetes, but also for those with other chronic illnesses using other kinds of self-management apps. This is because acceptability of such technology depends on finding a way to utilizing such apps to meet present patient needs and incorporate their use in a self-management fellowship with qualified professionals. However, this study had some limitations. A study design weakness could be that our sample comprised participants provided with a diabetes diary app for self-management purposes who were taken from a larger RCT. Although they were a homogeneous group, they had differing sociodemographic and clinical characteristics. They represented an adult group of all ages and both genders, each with a different disease history and condition, and therefore with different app requirement.

The focus of the participants’ experience was solely concerned with the use of a single app, and this might have provided less rich information in terms of answering of the research question. In addition, other sources of information such as login data and observational data could have given a richer contribution to our understanding of the app’s acceptability. The participants were provided with an HTC Corporation Microsoft Windows mobile smartphone. This was one of the early models; the screen was small, the ease of use was not the best, and smartphones were not widespread at the start of the RCT study. However, a strength of the study was that all participants had the same starting point. Moreover, factors related to technology and usability were the same for all, with diminished reliability owing to recall issues. This afforded us the opportunity to emphasize other aspects of acceptability such as utility and social references. Even though the smartphone could be difficult to manage, the app was developed in collaboration with persons with diabetes [40]. The possibility of automatic integration of blood glucose levels was at an early stage, even though the participants were required to enter diet- and activity-based information manually; far better systems are now available with wearables and sensors. Another strength in the study is that many of the participants had used the app for a year, which gave us data based on prolonged use.

Our data were not rich enough to be able to divide them between different health-care providers and to interpret different results for individualized treatment areas.

## Conclusions

In our study, we found that users’ acceptability of a mobile app for diabetes self-management differed, and we found both practical and social acceptability to be important at different levels. In the present study, we adapted Nielsen’s acceptability model according to these assumptions. If the app is used regularly, it could be useful in different ways, both supportive and educational. In contrast, it could turn out to be a burden requiring too much time, and not contributing to the efforts needed in changing lifestyles. From the perspective of social acceptability, we found some support from health-care personnel with diabetes knowledge parallel to the use of the app. The utility of the app for educational and supportive use could overcome the eventual lack of usability and establish its practical acceptability. We emphasize the need for raised awareness of vulnerable groups who could experience both digital and/or clinical distress beyond the intentions of the initiators of a mHealth intervention.

## Data Availability

Data are available from the corresponding author on reasonable request. The participants have received assurances of full confidentiality, and although we have removed identifying information, we cannot risk identification by making the interviews available for public inspection, due to the rules in the personal protection laws in Norway.

## Abbreviations

App: Application
COREQ: The Consolidated Criteria for Reporting Qualitative Research
EU: European Union
HbA1c: Glycated hemoglobin
MAST: The Model for Assessment of Telemedicine
mHealth: Mobile health
*P*: Participant
RCT: Randomized controlled study
REC: The Regional Committees for Medical and Health Research Ethics in Norway
RENEWING HeALTH: the European study REgioNs of Europe WorkINg toGether for HEALTH
